# The orthopaedic management of osteopetrosis in paediatric populations: a narrative review of the literature

**DOI:** 10.3389/fped.2026.1846893

**Published:** 2026-07-06

**Authors:** Antoine Chemtob, Elio Paris, Giacomo De Marco, Oscar Vazquez, Christina Steiger, Sana Boudabbous, Romain Dayer, Dimitri Ceroni

**Affiliations:** 1 Faculty of Medicine, University of Geneva, Geneva, Switzerland; 2 Paediatric Orthopaedics Unit, Paediatric Surgery Service, Geneva University Hospitals, Geneva, Switzerland; 3 Radiology Department, Geneva University Hospitals, Geneva, Switzerland

**Keywords:** bone deformities, deformity correction, fracture management, osteopetrosis, osteosynthesis, paediatric orthopaedics, pathological fractures

## Abstract

Osteopetrosis is a heterogeneous group of inherited bone disorders, where increased bone density and brittleness are associated with pathological fractures and osseous deformities. Treating patients with osteopetrosis is a significant challenge for orthopaedic surgeons. Altered bone characteristics affect the surgical care of fractures and impending fractures and complicate the correction of deformities, non-union and malunion. This review aims to summarise the existing literature on osteopetrosis and guide orthopaedic surgeons wishing to establish treatment protocols for paediatric patients.

## 1 Introduction

Osteopetrosis is a rare genetic disorder characterised by abnormally dense, brittle bone resulting from osteoclast dysfunction and an imbalance between bone formation and resorption (1). The condition is associated with an increased incidence of fractures and osseous deformities (2). Other features include bone marrow failure and anaemia, as well as neurological disorders due to nerve compression caused by exuberant bone growth (3).

Depending on the condition's severity, it is typically divided into three types, namely malignant infantile autosomal recessive osteopetrosis (ARO), intermediate autosomal osteopetrosis (IAO) and benign adult autosomal dominant osteopetrosis (ADO) (2, 4). The latter is divided into two subtypes (ADO I and II); ADO II is also known as Albers-Schönberg disease. Malignant infantile ARO affects just 1 in 250,000 children from birth, and the less severe adult ADO II has a prevalence of 1 in 20,000 adults (5, 6). All the forms show increased bone density due to impaired osteoclast-mediated bone resorption, but they differ in their severity, age of onset and their associated complications depending on the specific genetic defect present (7–9). Both sexes are affected, and no clear sex differences have been reported. Mutations affecting at least eight genes have been identified in the pathogenesis of osteopetrosis in humans (3). The prognosis differs markedly between subtypes: ADO tends to produce chronic morbidity with no major effects on overall survival, but without treatment, ARO is frequently fatal in infancy or early childhood. Occasional milder cases have been reported, nonetheless, with survival into adulthood reflecting the heterogeneity of the disease's expression (10).

Although paediatric orthopaedic surgeons play a key role in treating fractures and their complications, caring for osteopetrosis patients requires a multidisciplinary approach as the disease typically affects multiple organ systems (3). This review summarises historical and current protocols involving paediatric patients with osteopetrosis and seeks to guide orthopaedic surgeons tasked with diagnosing and treating this complex condition, while acknowledging that paediatric-specific orthopaedic evidence remains limited and is often extrapolated from adult or mixed-age cohorts.

## 2 Materials and methods

This narrative review is based on a comprehensive, non-systematic literature search performed in PubMed, Embase, and the Cochrane Library in December 2025, covering publications from January 1985 to December 2025. Search terms included combinations of osteopetrosis, pediatric, fracture, angular deformity, bone deformity, orthopedic, management, surgery, and osteosynthesis. This search strategy was supplemented by a manual review of the reference lists of all retrieved articles to identify additional relevant publications.

Studies eligible for inclusion were original articles investigating epidemiological, pathophysiological, genetic, histological, imaging, and therapeutic aspects of osteopetrosis. Prospective and retrospective cohort studies, case series, case reports, instructional lectures, and systematic or narrative reviews were included. Conference abstracts, animal studies, letters to the editor, and publications without an English abstract were excluded.

No restrictions were imposed on the mean age of the study population. Given the limited amount of paediatric-specific literature, studies involving adult populations were also included when they provided information relevant to the orthopaedic management of paediatric patients. Each article was reviewed independently by two reviewers (AC and EP) according to the eligibility criteria. In cases of disagreement, a third reviewer (DC) performed a rescreening and made the final decision. Findings were synthesized qualitatively to identify clinically relevant patterns regarding the diagnosis and orthopaedic management of osteopetrosis. Given the limited amount of paediatric-specific literature, adult and mixed-age studies were also considered when they addressed orthopaedic issues applicable to children, such as fracture fixation, delayed union, deformity correction or implant-related complications.

## 3 History of diagnosis

The discovery of osteopetrosis was closely correlated with the development of radiology. In 1904, Albers-Schönberg, a gynaecologist and radiologist, first described osteopetrosis in a 26-year-old male who presented with a pathological fracture of the femur (11). Due to the bone's opaque radiological aspect, its widened cortices and dramatically decreased medullary cavities, the condition was first described as *marble bone disease*. The radiographic criteria of malignant osteopetrosis were defined by Sick in 1914 (12). Nine years before that, Alexander reported the case of a 43-year-old female who presented with multiple fractures, dental injuries, anaemia and partial facial palsy. Further findings supported the idea that the disease followed an autosomal recessive inheritance pattern (13).

The term *osteopetrosis* (from the Greek *osteon*, meaning bone, and *petros*, meaning stone) was introduced in 1926 by Karshner, replacing marble bone disease since skeletal fragility resembled limestone more than marble (14). By 1936, when Wortis described how the medullary cavity could be entirely filled with endochondral bone, leaving insufficient space for haematopoietic cells and resulting in bone-marrow aplasia, the idea that osteopetrosis was a systemic condition gained traction (15). In 1938, Nussey described how some bone biopsies showed a complete absence of osteoclasts while others showed a high number of enlarged multinucleated osteoclasts, the latter being associated with sclerotic cancellous bone and, sometimes, deficient mineralisation of the bone matrix (16). These observations contributed to establishing the correlation between histopathological findings and disease severity. The same year, going beyond the recognised lethal autosomal recessive form of the disease, Nussey also described a milder manifestation of osteopetrosis “handed down directly from generation to generation” (17). In 1965, Dent et al., showed that even some qualitative defects in osteoclasts could suffice to generate a mild form of osteopetrosis (18). This observation provided evidence that the disease was expressed as both a lack of osteoclasts and osteoclasts with functional abnormalities.

## 4 Etiopathogenesis

Bone remodelling is a continuous yet precisely regulated bodily process involving a delicate balance between bone formation by osteoblasts and bone resorption by osteoclasts (19, 20). Transcription factors and cytokines regulate the formation, maturation and functions of those cells. Disturbances to these factors can alter the balance and result in osteopetrosis if bone formation exceeds bone resorption. Three primary mechanisms responsible for the generation of osteopetrosis have been identified: two quantitative and one qualitative (21–23). The most important is the complete failure of mononuclear preosteoclast formation. The second contributing mechanism involves a defect in the fusion and polarisation process that generates mature multinucleated osteoclasts. The third, altered osteoclast function, participates in the pathogenesis of osteopetrosis (24).

Biallelic mutations of the TCIRG1 gene—responsible for encoding a subunit of the vacuolar H+-ATPase proton pump on cell membranes—account for approximately half of ARO cases. Mutations of the CLCN7 chloride channel gene—responsible for pH regulation in osteoclast cells—are responsible for 10%–15% of cases. Other described mutations involve the OSTM1, SNX10 or PLEKHM genes, all of which regulate crucial osteoclast functions. Osteoclasts are present, but they cannot function due to impaired acidification or vesicle trafficking, a condition called osteoclast-rich ARO. In contrast, osteoclast-poor ARO results from a failure of osteoclast differentiation associated with mutations of the TNFSF11 (RANKL) or TNFRSF11A (RANK) genes (7, 8, 25).

Heterozygous or homozygous mutations in the CLCN7 and PLEKHM1 genes can also result in IAO (9). Other genes involved include CAII, which is responsible for producing carbonic anhydrase II, an enzyme that enables the reabsorption of bicarbonate ions in the kidney. IAO is associated with an increased incidence of fractures and anaemia in children, but without the fatal bone marrow failure seen in ARO (8).

Seventy-five percent of ADO cases are associated with mono-allelic mutations of the CLCN7 gene. More rarely, ADO results from missense mutations in the TCIRG1 gene (8, 26, 27). ADO is typically a milder form of the disease and can be an incidental finding in adolescent or adult patients presenting with a fracture, scoliosis or osteomyelitis. In some cases, diagnosis remains clinical as genetic testing often fails to identify the genetic mutations involved (28).

Table 1 summarises the principal genes involved in osteopetrosis, their associated clinical subtypes, pathogenic mechanisms and typical clinical presentations.

**Table 1:** Main genetic forms and clinical characteristics of osteopetrosis.

Gene	Osteopetrosis subtype	Main pathogenic mechanism	Typical presentation
TCIRG1	ARO	Defective osteoclast acidification (V-ATPase proton pump)	Severe infantile disease with bone marrow failure, fractures and cranial nerve compression
CLCN7	ARO, IAO, ADO	Defective chloride transport and pH regulation in osteoclasts	Highly variable phenotype ranging from severe infantile disease to mild adult forms
OSTM1	ARO	Impaired osteoclast function	Severe infantile disease often associated with neurological involvement
SNX10	ARO	Defective vesicle trafficking	Severe infantile osteopetrosis with fractures and growth impairment
PLEKHM1	ARO, IAO	Defective vesicle trafficking	Intermediate phenotype with recurrent fractures and deformities
TNFSF11 (RANKL)	ARO	Failure of osteoclast differentiation	Osteoclast-poor osteopetrosis with severe skeletal manifestations
TNFRSF11A (RANK)	ARO	Failure of osteoclast differentiation	Osteoclast-poor osteopetrosis with severe skeletal manifestations
CA2	IAO/ARO with renal tubular acidosis	Carbonic anhydrase II deficiency	Fractures, anaemia, renal tubular acidosis and possible cerebral calcifications, without severe bone marrow failure

## 5 Epidemiology

Due to its rarity, the overall prevalence and incidence of osteopetrosis are difficult to estimate. Infantile malignant ARO has an incidence of 1 in 250,000 births, and ADO II (or Albers-Schönberg disease) affects 1 in 20,000 adults (3, 29). The incidences of IAO and ADO I remain unknown due to their extraordinary rarity.

There seem to be no sex differences, but specific types of osteopetrosis, such as its very rare X-linked form (few cases have been reported), are expected to occur predominantly in males due to its mode of inheritance. Penetrance can be incomplete, ranging from 60% to 90% in families with ADO (30), and the affection can skip generations. The proportion of cases caused by *de novo* mutations has not been reported. Disease severity can vary significantly within families due to incomplete penetrance and variable expressivity (31).

## 6 Natural history and clinical presentation

The age at presentation, severity and natural history of osteopetrosis vary considerably depending on the disease's genetic subtype. In the absence of modern therapeutic interventions—such as haematopoietic stem cell transplantation (HSCT) or targeted molecular therapies—the disease follows a progressive course characterised by skeletal fragility, systemic complications and, in severe forms, reduced survival. The milder form, ADO, follows a chronic trajectory, and the age of onset is typically in young adulthood (32). Longitudinal studies have demonstrated that skeletal complications accumulate over time, with fractures reported in 84% of cases, osteomyelitis in 16%, eyesight loss in 19% and bone marrow failure in 3%. Complications that appear during childhood lead to progressively worsening skeletal deformities and reduced quality of life (30). Despite significant morbidity, life expectancy is usually preserved, and early mortality is uncommon in ADO. In contrast, ARO is associated with rapid progression and a poor prognosis. Early mortality is typically due to the failure of intramedullary haematopoiesis in a context involving medullary obstruction. This leads to extramedullary haematopoiesis and hepatosplenomegaly. Bone marrow failure also leads to anaemia, thrombocytopenia and leukopenia, and very young patients affected by ARO are often dependent on frequent blood transfusions. Thrombocytopenia can lead to severe bleeding that can be fatal when intracranial. Patients suffering from leukopenia are prone to recurrent infections, such as chronic sinusitis or pneumonia. Phenotypical features found among ARO patients also include club-shaped long bones, frontal bossing and proptosis associated with thickening of the eyes' orbital walls (33, 34). Patients are prone to dental problems, including delayed eruption and tooth malformations. The narrowing of nerve foramina may cause hydrocephalus, deafness, blindness and other palsies. Sclerosis of the base of the skull may affect neurological and otolaryngological function, and patients can present with choanal stenosis, obstructive sleep apnoea and feeding issues (35). Severe mandibular osteomyelitis is associated with poor vascularisation of the jaw (4, 36, 37). Patients with ARO also present with symptoms of hypocalcaemia, such as tetanic seizures or secondary hyperparathyroidism, as they cannot mobilise calcium reserves because the number and activity of the osteoclasts in their bone is so low (38).

Clinically, ARO patients typically present with recurrent fractures, growth impairment, cranial nerve compression leading to neuropathies, hypocalcaemia that can provoke tetanic seizures, and severe pancytopenia during infancy. In some patients, additional features—neurodegeneration, developmental delay, skin or immune abnormalities, or renal tubular acidosis—suggest rarer genetic variants. ADO, however, usually manifests later in childhood or adolescence, predominantly involving skeletal problems including fractures and osteomyelitis (3).

## 7 Radiological investigations

Because investigations using radiological imaging frequently produce highly pathognomonic evidence of osteopetrosis, they have taken on a predominant and essential role in establishing and confirming the disease's diagnosis and in identifying the classic sites of skeletal involvement (39, 40). In severe forms, conventional radiology often proves quite sufficient for providing a rapid confirmation of the suspected diagnosis (40). In milder forms, imaging findings can be the first or even the only sign of the disease (41). A variety of imaging techniques can be used to assess the different features of osteopetrosis throughout the skeleton, but especially for evaluating complications. It is commonly accepted that conventional radiography can identify the main, well-known signs of osteopetrosis, starting with the classic sign of abnormally increased bone density. This can also be appraised using dual-energy x-ray absorptiometry, especially in milder forms of the disease.

**Figure 1:**
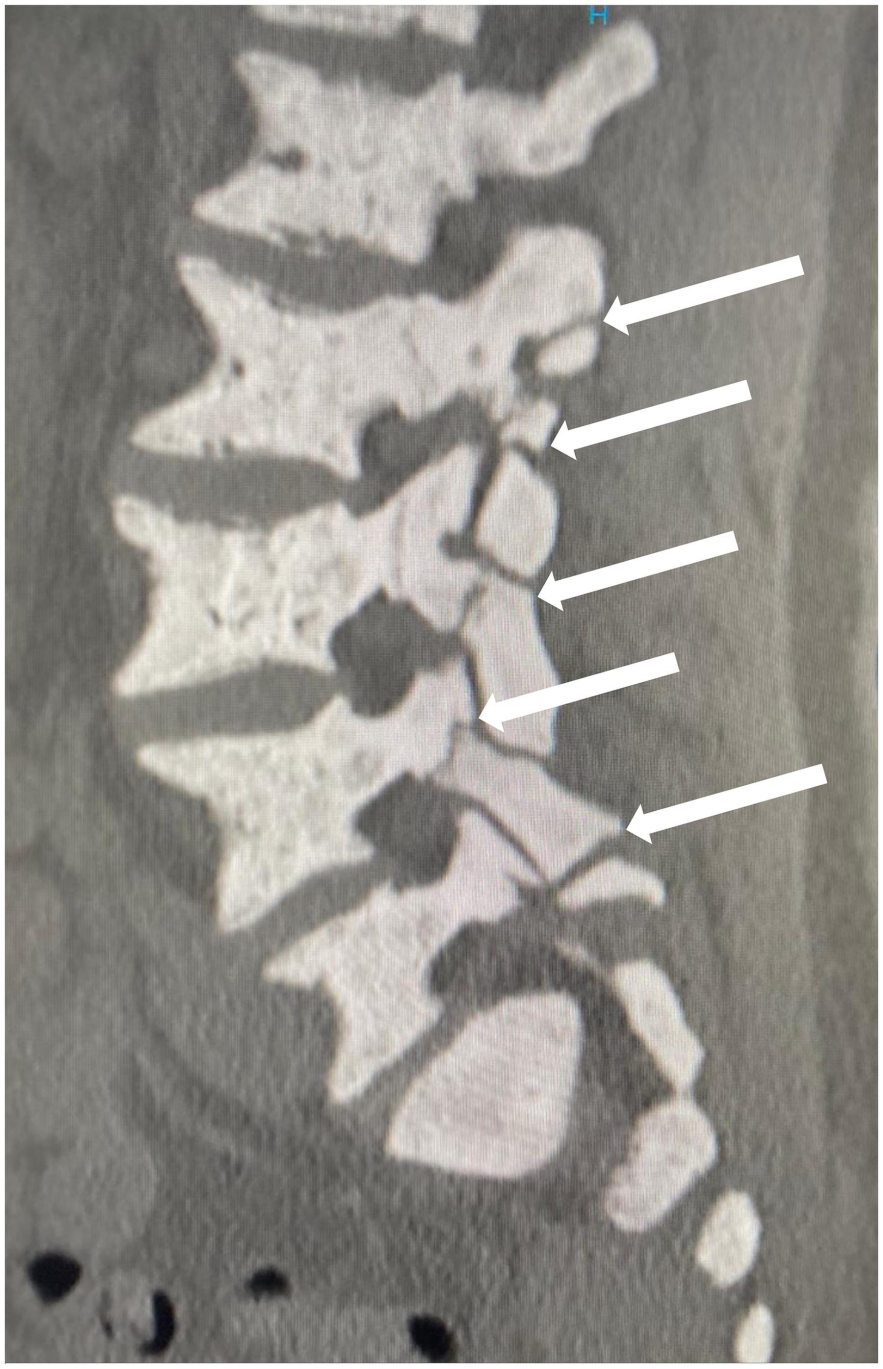
Midline sagittal CT scan image of the lumbar spine showing pars interarticularis defects (white arrows) at multiple levels of the lumbar spine.

Alongside conventional radiography, computed tomography (CT) can also identify numerous signs of osteopetrosis. It is a more discriminating tool, however, providing both greater detail and higher spatial resolution. CT can be helpful as it is better at detecting potentially associated fractures, particularly in the posterior neural arch of vertebrae (Figure 1), or cases of inflammatory bone diseases. Magnetic resonance imaging (MRI) can also provide other essential indications in the evaluation of patients with osteopetrosis. It can be very useful for evaluating the amount of bone marrow space remaining in the most severely affected patients. Additionally, MRI of the brain and eye sockets can be used to assess any narrowing of the auditory nerve (eighth cranial nerve), compression of other cranial nerves (notably the optic nerve) due to bone thickening, and narrowing of the foramina of central nerve channels that can result in nerve compression and neuropathic changes.

In addition to these skeletal and neurological evaluations, abdominal ultrasound can be used to detect hepatosplenomegaly, a common sign of extramedullary haematopoiesis in patients with compromised bone marrow function.

For every clinical suspicion of osteopetrosis or incidental radiographic finding, Watson et al. (42) recommended conducting a protocolled radiographic survey of the skeleton, including:

- anterior–posterior (AP) and lateral projections of the skull (lateral view should include the jaw to assess the mandibular angle, which is absent in cases of pycnodysostosis (43);
- lateral projections of the thoraco-lumbosacral spine (+/- the cervical spine in younger infants and neonates);
- AP views of the chest;
- AP projections of the pelvis;
- AP projections of one upper limb (right or left);
- AP projections of one lower limb (right or left);
- dorso-palmar projections of the left hand.

**Figure 2:**
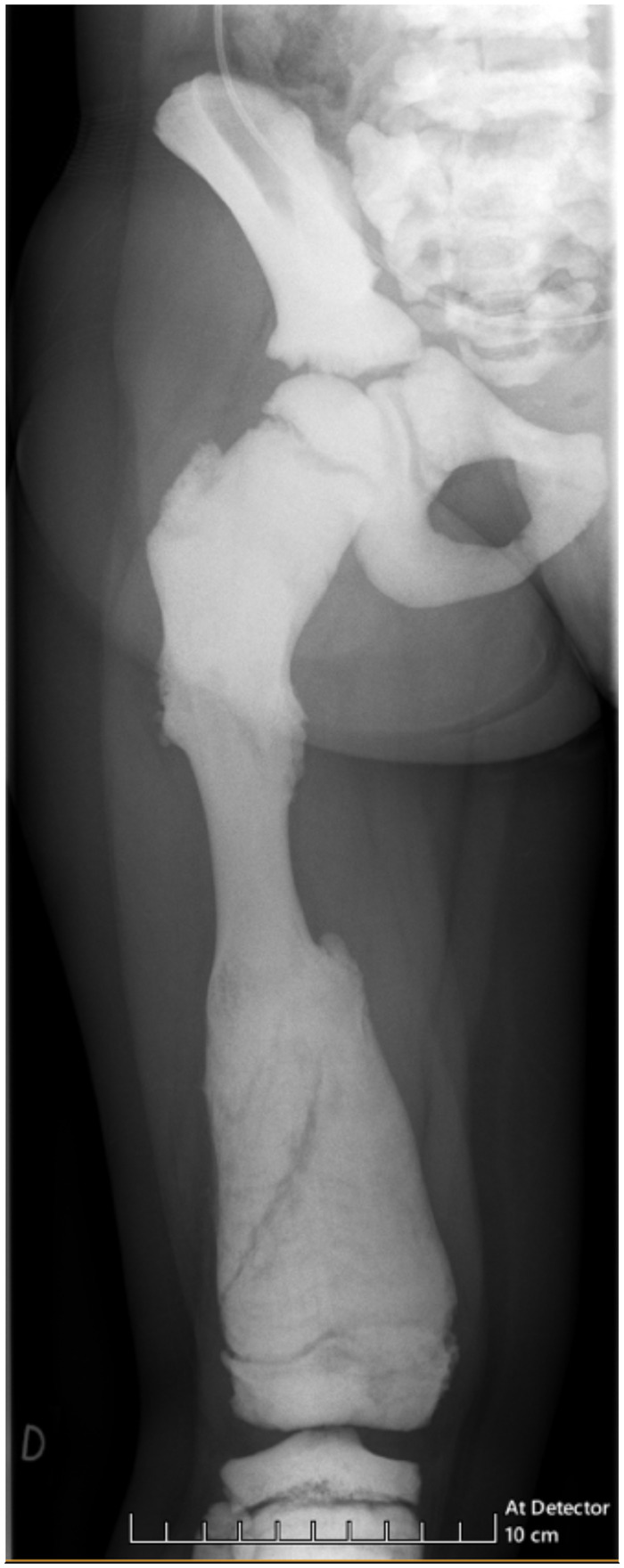
AP radiograph of the right femur of a 10-year-old patient with ADO II. It reveals the typical flask-shaped deformity of the distal femoral metaphysis.

A thorough review of these images should enable the identification of several radiographic patterns characteristic of osteopetrosis. Identifying these features is crucial for confirming the diagnosis and orienting further genetic and clinical evaluations. The central feature of osteopetrosis is the generalised bone sclerosis that led to the disease's historical name of marble bone disease. As well as bone sclerosis, another typical finding is a *bone within a bone* appearance, most commonly found in fingers, toes, vertebrae, iliac wings and long bones (40). This appearance results from the defective osteoclastic resorption of the calcified cartilage matrix, leading to abnormally thick, dense bones with a narrowing of the marrow cavities (44). The same remodelling defect also explains the development of the Erlenmeyer flask deformities caused by a failure of normal metaphyseal modelling, which usually converts the broad metaphysis into a narrower diaphysis. In osteopetrosis, this transition is incomplete, leaving an under-modelled metaphyseal region that merges into the diaphysis. The deformity is most prominent in the distal femur, where it appears as a wide metaphysis with a characteristic flask-like contour (Figure 2).

**Figure 3:**
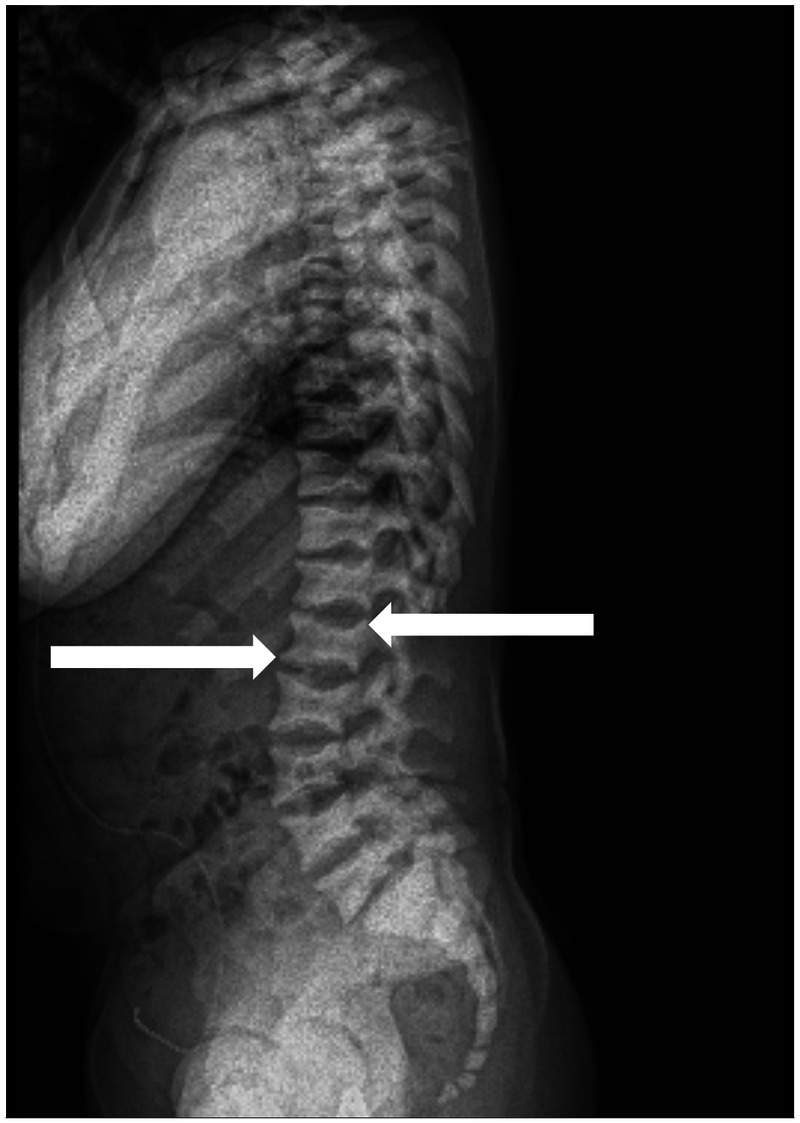
Lateral radiograph of a 23-year-old patient with OP (ADO II), showing the hyperdense sclerotic superior and inferior endplates of the vertebral bodies (white arrows), also termed “sandwich vertebrae”.

The spine is also affected; due to defective bone remodelling, vertebral bodies accumulate depositions of dense, sclerotic bone along their superior and inferior endplates, with a relatively lucent or less sclerotic central portion (45). This generates a typical *sandwich vertebrae* or *rugger jersey* appearance on radiographs (Figure 3). Clinically, this finding is a radiological hallmark of milder forms of osteopetrosis, such as ADO II or IAO.

**Figure 4:**
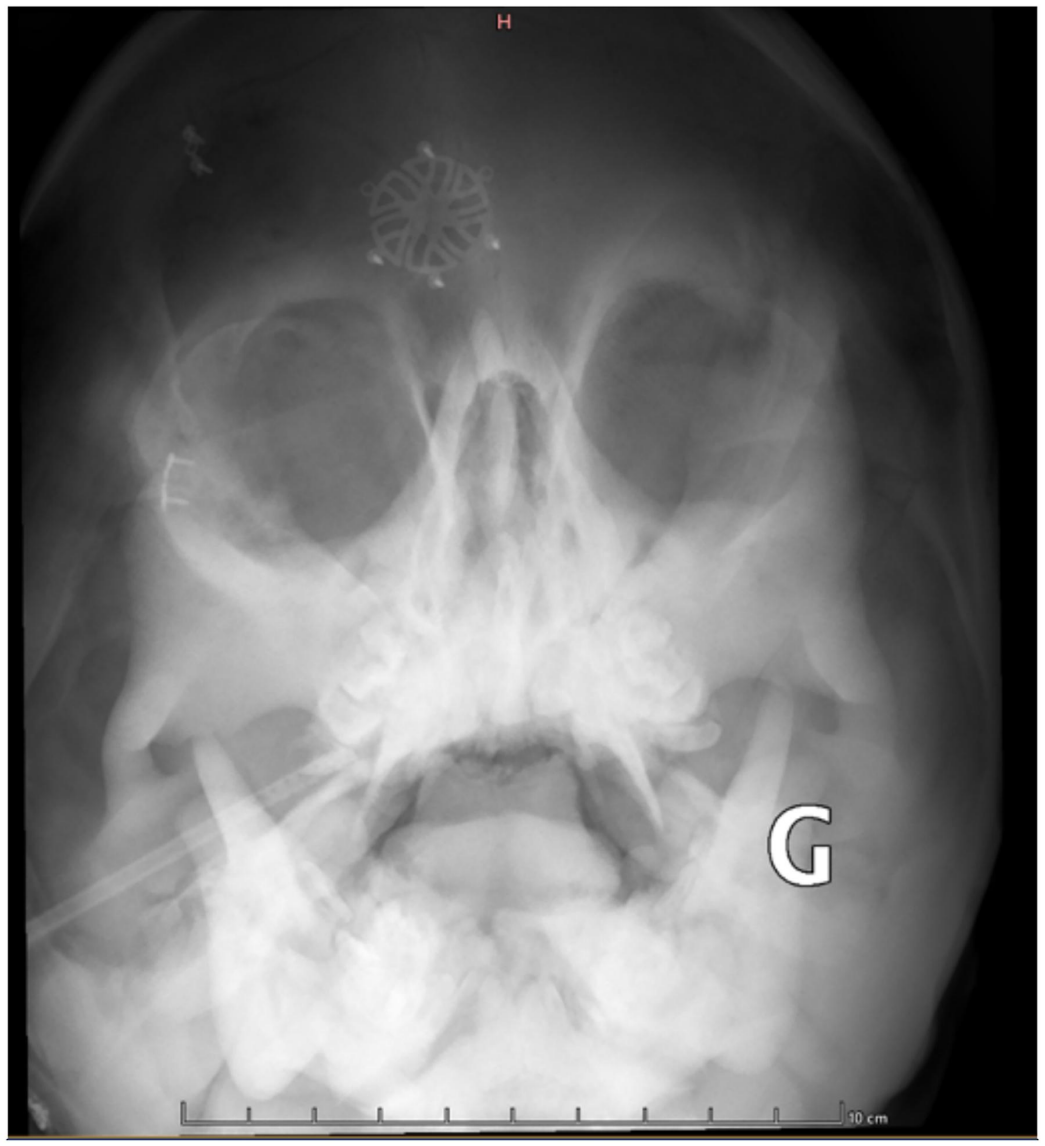
Blondeau projection radiograph of the skull of a 10-year-old child with OP, showing typical skull base thickening.

Thickening of the skull's base can drive some severe neurological complications, but it can easily be seen on lateral and Blondeau (Figure 4) projections. Nonetheless, a CT examination of the foramina's degree of stenosis, combined with MRI to visualise nerve damage, is mandatory before treating the patient. An early assessment of any affected nerves is key to referring the patient for an HSCT, as we discuss below. Twice-yearly checkups with an ophthalmologist are also essential (46).

## 8 Genetic testing

Genetic testing is now essential to the early and targeted management of osteopetrosis. Radiographs may clearly reveal a condition causing the patient's bones to exhibit the characteristics previously described, but geneticists must be involved without delay, as the relevant mutation determines the disease's aggressiveness. It should not be forgotten that ARO can cause significant mortality in the first year of life and that numerous complications—such as deafness and visual impairment—can be avoided through prompt transfusions and HSCT (8). Next-generation sequencing, whether through whole exome sequencing or targeted gene panels, is now used routinely, enabling rapid analysis, accurate molecular diagnosis, and thus better care (47). These modern techniques for sequencing candidate genes are faster than the classic Sanger method, and continuous improvements in sequencing are making genetic testing faster, cheaper and more widely available. Nevertheless, in some cases, the precise pathogenetic mechanism remains unclear. Despite this, the constantly increasing amount of data available in the literature makes it possible to suspect which type of defect is affecting the patient's osteoclasts (48).

## 9 Orthopaedic management of osteopetrotic lesions

Orthopaedists should be aware of osteopetrosis and its signs and symptoms since the disease elicits a high lifetime risk of fractures, with estimates ranging from 40% to 50%. Long-bone fractures are very common in osteopetrosis (49), with a lifetime prevalence of 1–10 fractures per patient with the disease's congenital form (50). Even if bone healing can occur, the bone's increased density, its decreased vascularity and disturbances to the bone remodelling process can impair it, making fractures more prone to slower healing and subsequent non-union. The actions of orthopaedic surgeons can, therefore, positively affect the outcomes of these fractures.

However, successfully managing osteopetrosis remains a significant challenge for paediatric orthopaedic surgeons: its vast clinical spectrum involves a variety of bone segments, and, above all, it has a huge impact on bone quality and stability. The orthopaedic treatment of osteopetrosis principally seeks to manage uncontrollable bone pain, care for impending pathological fractures, correct deformities, manage the occurrence of degenerative osteoarthritis, and promptly recognise and treat any osteomyelitis. There is thus a host of concepts and principles regarding osteopetrotic bones that paediatric orthopaedists must keep in mind before embarking on treatments.

**Figure 5:**
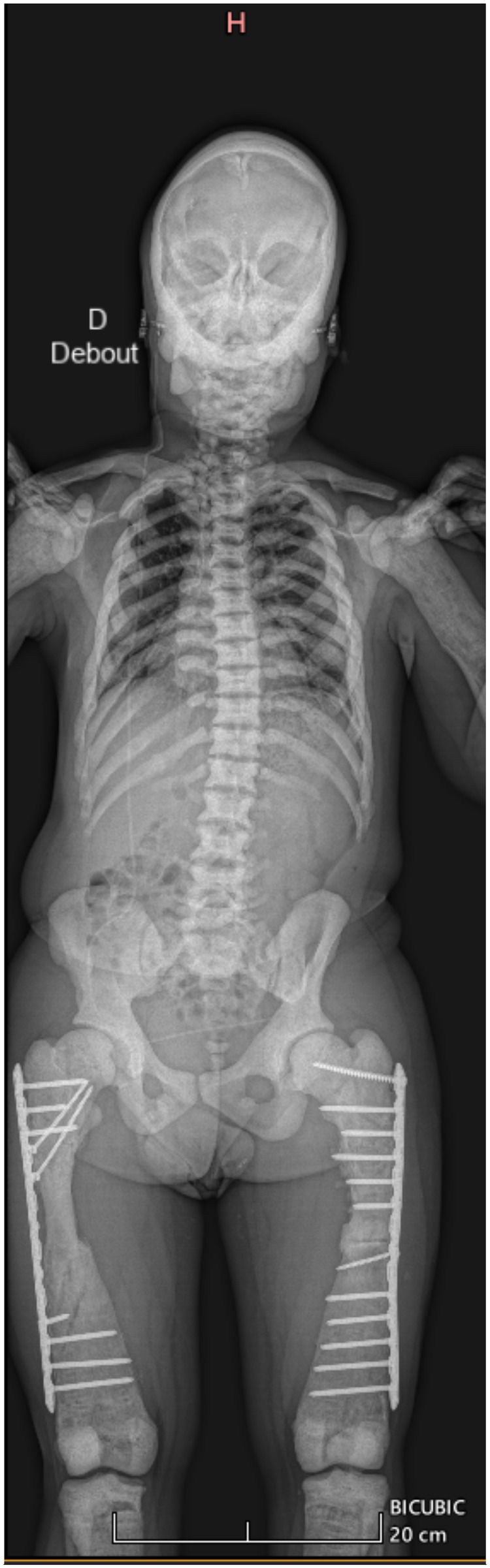
AP projection radiograph of a 22-year-old patient, 3 months after a right femur osteotomy with axis correction and right femur osteosynthesis (plate and screws).the patient's right lower limb reveals the previous year's procedure: the open reduction of an intertrochanteric fracture, fixed with double screws, and percutaneous minimally invasive osteosynthesis of the right femur (curved femoral plate with 13 holes, 247 mm).

1. It is essential to remember that there can be no spontaneous resolution of osteopetrosis, just as there is no definitive cure. Bone marrow transplantation is currently a good treatment option for severe infantile osteopetrosis since it can improve but not normalise bone remodelling and can contain the rate of complications associated with the disease.
2. It is important to keep in mind that the characteristics of the osteopetrotic bone itself can affect even a well-prepared treatment. Despite high bone mass, osteopetrosis results in brittle bones that are susceptible to fracturing during surgery; because the structure is abnormal, it is less resistant to mechanical stress.
3. Bone healing is disturbed. It is recognised that impaired bone remodelling is associated with the decreased vascularity that negatively impacts bone healing (51). The healing process will thus be hindered by the body's inability to properly remodel bone, the process during which old bone is resorbed and replaced by new bone. It follows, therefore, that even if bone healing remains possible, it will typically take longer in patients with osteopetrosis than in individuals without the disease. Indeed, we should expect delayed consolidation or non-union to be far more probable among patients with osteopetrosis, especially at certain fracture locations (52).
4. The concepts set out above mean that angulated fractures cannot be remodelled and, when present, deformities may even give rise to recurrent fractures and thus to the further accentuation of those deformities (52). Any residual deformities should be avoided as much as possible and, when present, they should be corrected at all costs.
5. Since genetic testing can identify the gene variant in over 90% of osteopetrosis patients, the indications for performing a bone biopsy to confirm the diagnosis have diminished notably. Bone biopsies are now exceptional, not routine, constituting an invasive procedure with inherent risks (53).
6. Paediatric orthopaedists will mainly be confronted with pathological fractures of the long bones. Fractures in osteopetrotic bones generally occur at a right angle to the cortex, and they thus usually appear in a transverse or short oblique pattern, often with minimal displacement (2, 54, 55).
7. There is currently no unambiguous consensus on whether conservative or surgical treatments should be adopted for fractures in patients with osteopetrosis (56). However, opting for non-operative treatment regimens is considered legitimate for children, adolescents and most patients presenting with upper-limb fractures (57). Regarding other lesions, such as femoral intertrochanteric, subtrochanteric and femoral neck fractures, that can progress to delayed bone union, non-union or malunion, surgical treatment is likely to be associated with a more favourable prognosis (57).
8. Osteosynthesis using plates is the recommended treatment for most lower-limb fractures, despite the risk of failure (58). Plate fixation circumvents the inherent problems of the absence or narrowing of the intramedullary bone canal that characterise this condition. However, using conventional plates reveals the specific mechanical problems inherent in the areas under stress at the plates’ ends, increasing the risk of fractures distally or proximally to the plate material. Due to their rigidity, plates themselves can be prone to fracture because of the high stresses to which they are subjected during the delayed consolidation process. To overcome these complications (fractures occurring at the plate's end or affecting the material itself), locked titanium plate implants represent an interesting new option as their reduced rigidity is less likely to result in breakages (59). An example of deformity correction and fixation is shown in Figure 5. To avoid fractures around the plates, some authors have recommended using a unicortical screw for the last screw, as well as inserting it in an oblique direction, away from the fracture site, in order to redistribute the stresses at the end of the plate (58).
9. Since the bones affected by osteopetrosis have either no medullary canal or their medullary cavity is significantly composed of endochondral bone, intramedullary fixation may be particularly challenging. Indeed, the intra-operative challenges associated with locating the medullary canal, difficult reaming, and heat generation during that reaming, further complicate the procedure. Case reports have nonetheless shown that the intra-medullary nailing of osteopetrotic bone is feasible, although technically difficult, with meticulous planning and appropriate techniques. Intramedullary fixation should be considered for proximal femoral fractures because of its superior biomechanical performance under axial loading.
10. Surgeons must therefore adapt their operating techniques to the particular bone's characteristics. When considering drilling a hard bone, a high-density metal drill, a diamond drill bit or a tungsten carbide drill is recommended (60). Moreover, it is essential to have multiple drill bits available in progressively larger sizes and to regularly withdraw and clean the drill and the grooves drilled. Some authors also recommend controlled drilling in short, spaced cycles, using low-speed, high-torque systems when available, to reduce drill-bit breakage and avoid uncontrolled toggling (61–63).
11. In parallel with these mechanical precautions, using powerful, high-speed motors appears to be crucial to avoiding back-and-forth drilling. The bone overheating responsible for thermal necrosis (64) can be prevented by continuous irrigation with cool saline solution and managing pauses so that drilling never exceeds 30 s. Drilling for longer generally results in temperatures ranging between 47 °C and 55 °C, which leads to bone cell death (65–67).

## 10 Pharmacological treatments

Transplanted haematopoietic stem cells that generate osteoclasts are currently the only available or even envisaged curative therapy. Unfortunately, not all forms of the disease can be cured using this treatment; potentially eligible patients are selected based on their mutation. They must have a mutation that significantly affects osteoclast function, as HSCT has severe side effects. The treatment is reserved for ARO forms of osteopetrosis because the risks of transplantation are too high for ADO patients, who typically have a non-lethal, although very debilitating, form of the disease. Note that HSCT can reverse visual impairment if treatment occurs before the child is 3 months old (68), underlining the need for early diagnosis. Not every mutation causing ARO can be corrected using HSCT, however, including mutations of the RANKL or OSTM1 gene. In RANKL-related forms, the defect lies outside the haematopoietic lineage, preventing osteoclast differentiation and activation, despite transplantation. In OSTM1 mutations, primary neurodegeneration remains fatal even if osteoclastic function is restored (69).

Interferon gamma (IFN *γ*) treatment can be proposed as a bridging therapy, giving the patient a chance of survival while awaiting a matched donor for HSCT. This is not a curative option, but a study by Key et al. (70) showed that it lowered trabecular bone density, increased marrow space, augmented mean haemoglobin concentrations and improved superoxidation (therefore enhancing the bone's ability to fight infection).

Unfortunately, there is no approved cure for dominant osteopetrosis. For both ADO and ARO, treatment remains largely supportive rather than curative. Supportive measures include blood transfusions in cases of bone marrow failure and orthopaedic interventions aimed at stabilising fractures (technically challenging and associated with a high risk of post-operative infection). Other symptomatic treatments include using analgesics to manage bone pain and antibiotics to combat osteomyelitis (71).

Other pharmacological treatments have been attempted in the past, including parathyroid hormone, vitamin D3 and calcium supplements—none demonstrated efficacy (67, 68). We still need to deepen our understanding of the pathophysiology of osteopetrosis and the involvement of each gene mutation. Gene therapies may provide new potential curative treatments in the future, particularly as treatment response is increasingly understood to be mutation-dependent.

## 11 Conclusions

Osteopetrosis is a rare genetic disorder caused by defective bone resorption and resulting in bone fragility due to diffuse generalised bone sclerosis. Different forms of the disease (malignant infantile autosomal recessive osteopetrosis, ARO; intermediate autosomal osteopetrosis, IAO; benign adult autosomal dominant osteopetrosis, ADO) cover a large spectrum of severity, ranging from a fatal form in the neonatal period to a benign form in adults. This diversity is due to genetics, with different mutations explaining the disease's phenotypic variation. Precise diagnoses are based on clinical findings and specific radiological characteristics. Technological advances in genetics (particularly next-generation sequencing) have revolutionised the classification and management of the disease. Early diagnosis (including prenatally when there is a family history) improves survival in severe cases that may otherwise be fatal in the first months of life. Haematopoietic stem cell transplantation remains the only curative treatment and is reserved for the most severe cases. Pharmaceutical treatments are limited and mainly supportive in nature. Since bone fractures are predominant in this disease, the orthopaedist's role and their understanding of osteopetrotic bone are key to preventing pre- and post-operative complications. As with all rare and multisystemic diseases, multidisciplinary care is crucial. It must include paediatricians, haematologists, radiologists, metabolic bone specialists, pathologists, geneticists, ophthalmologists, neurosurgeons, otolaryngologists, infectious diseases specialists, physiotherapists and orthopaedists. Advances in genomics and gene therapies are paving the way for new perspectives in the realm of personalised treatment. Until these advances are well established, orthopaedists remain key players because children with osteopetrosis require anticipation of impaired remodelling, delayed union, deformity progression and fixation-related complications.
